# Behavioral and ERP Correlates of Long-Term Physical and Mental Training on a Demanding Switch Task

**DOI:** 10.3389/fpsyg.2021.569025

**Published:** 2021-02-23

**Authors:** Pablo I. Burgos, Gabriela Cruz, Teresa Hawkes, Ignacia Rojas-Sepúlveda, Marjorie Woollacott

**Affiliations:** ^1^Department of Neuroscience, Universidad de Chile, Santiago, Chile; ^2^Department of Physical Therapy, Universidad de Chile, Santiago, Chile; ^3^Institute of Neuroscience and Psychology, University of Glasgow, Glasgow, United Kingdom; ^4^Oregon Research Institute, Eugene, OR, United States; ^5^Department of Human Physiology and Institute of Neuroscience, University of Oregon, Eugene, OR, United States

**Keywords:** executive function, switching, EEG sources, ERP, physical-mental practice

## Abstract

Physical and mental training are associated with positive effects on executive functions throughout the lifespan. However, evidence of the benefits of combined physical and mental regimes over a sedentary lifestyle remain sparse. The goal of this study was to investigate potential mechanisms, from a source-resolved event-related-potential perspective, that could explain how practicing long-term physical and mental exercise can benefit neural processing during the execution of an attention switching task. Fifty-three healthy community volunteers who self-reported long-term practice of Tai Chi (*n* = 10), meditation + exercise (*n* = 16), simple aerobics (*n* = 15), or a sedentary lifestyle (*n* = 12), aged 47.8 ± 14.6 (SD) were included in this analysis. All participants undertook high-density electroencephalography recording during a switch paradigm. Our results indicate that people who practice physical and mental exercise perform better in a task-switching paradigm. Our analysis revealed an additive effect of the combined practice of physical and mental exercise over physical exercise only. In addition, we confirmed the participation of frontal, parietal and cingulate areas as generators of event-related-potential components (N2-like and P3-like) commonly associated to the performance of switch tasks. Particularly, the N2-like component of the parietal and frontal domains showed significantly greater amplitudes in the exercise and mental training groups compared with aerobics and sedentary groups. Furthermore, we showed better performance associated with greater N2-like amplitudes. Our multivariate analysis revealed that activity type was the most relevant factor to explain the difference between groups, with an important influence of age, and body mass index, and with small effects of educational years, cardiovascular capacity, and sex. These results suggest that chronic combined physical and mental training may confer significant benefits to executive function in normally aging adults, probably through more efficient early attentional processing. Future experimental studies are needed to confirm our results and understand the mechanisms on parieto-frontal networks that contribute to the cognitive improvement associated with practicing combined mental and aerobic exercise, while carefully controlling confounding factors, such as age and body mass index.

## Introduction

Research studies on the benefits of physical and mental training have shown a positive effect on cognitive function throughout the lifespan (Kramer et al., 1999; Chan and Woollacott, 2007; Weinstein et al., 2012; Hawkes et al., 2014a). Many types of exercise have been studied: golf (Shimada et al., 2018), dance, (Chuang et al., 2015; Eggenberger et al., 2015), aerobic and resistance training (Kramer et al., 1999; Lucas et al., 2012; Weinstein et al., 2012; Chapman et al., 2013; Steinberg et al., 2015; Tsai et al., 2018; Wu et al., 2019), Tai Chi (Matthews and Williams, 2008; Hawkes et al., 2014a) and the mental training required for meditation (Chan and Woollacott, 2007; Moore and Malinowski, 2009; Zeidan et al., 2010; Moynihan et al., 2013; Elliott et al., 2014; Raichlen and Alexander, 2017).

Though research studies on the individual effects of physical and mental training report improvement in cognitive function, research on combined physical and mental training—such as Tai Chi, dancing, sports, and other exercise disciplines that combine the simultaneous practice of cognition + moderate exercise—suggests increased benefits compared to exercise that does not require attention, planning, memory or other cognitive challenge (Matthews and Williams, 2008; Voss et al., 2009; Anderson-Hanley et al., 2012; Maillot et al., 2012; Hawkes et al., 2014a; Wayne et al., 2014; Zhu et al., 2016; Raichlen and Alexander, 2017). Evidence for cognitive improvement resulting from combined mental and physical training in humans is mainly behavioral considering different kinds of cognitive challenges and the simultaneous or delayed performance of physical and cognitive practice (Anderson-Hanley et al., 2012; Barcelos et al., 2015; Eggenberger et al., 2015). Also, there is evidence that the combined regimes have advantages over the physical but not over a pure cognitive training (Raichlen and Alexander, 2017). Thus, more research is needed to elucidate how physical and mental exercise affects the neural processes underlying cognitive function (Bamidis et al., 2014; Raichlen and Alexander, 2017).

The current study is a secondary data analysis of data collected from a cross-sectional observational study that evaluated differences in cardiovascular and executive attention metrics across groups self-reporting adherence to different health regimes (Hawkes et al., 2014a,b)—Tai Chi, meditation + exercise, and simple aerobics (e.g., walking, jogging). A sedentary control group was included. Hawkes et al. (2014a) reported that Tai Chi and meditation + exercise practitioners had statistically faster reaction times, lower local switch costs, and greater P300 event-related-potential (ERP) amplitudes compared to sedentary controls, but not the aerobic practitioners, who performed similar to the sedentary controls. These results are in line with the previous literature; there is evidence for larger P3b amplitudes in elderly adults who practiced moderate aerobic exercise compared to sedentary elderly controls (Hillman et al., 2004). Hillman et al. (2006, 2003) also reported larger P3b amplitudes and shorter latencies on a task-switching paradigm in active versus inactive young and older adults. Meditation studies have also shown increased ERP amplitude or decreased latency during tasks requiring attentional focus and distractor inhibition (Slagter et al., 2007). Van Leeuwenn et al. reported larger N1, N2, P1, and P3 ERP amplitudes in meditators compared to controls during an attentional task (van Leeuwen et al., 2012). Larger ERP amplitudes may index more attentional resources allocated during the updating of working memory, and shorter latencies may index more efficient neural processing (Hillman et al., 2006, 2003).

Several advances in the study of source brain activity (Onton et al., 2006; Makeig and Onton, 2011; Akalin Acar et al., 2013; Bigdely-Shamlo et al., 2013) allow separate weighted-combinations of mean event-related activities arising from several to many cortical sources that are mixed on scalp ERPs channels. Therefore, the aim of the present study was to investigate potential mechanisms, from a source-resolved ERP perspective, that could explain how practicing long-term physical and mental exercise can benefit neural processing during the execution of an attention switching task. In addition, we performed an analysis of the lifestyle and demographic factors of the groups in relation to the switching performance and the associated ERP activity that could also contribute to the group differences observed.

We hypothesized that this new source analysis would reveal health regime differences on source-ERPs with probable source in the prefrontal, parietal and anterior cingulate cortex, as previous functional magnetic resonance imaging (fMRI) studies have shown the relevance of these areas for improved attentional and executive function in both meditators and Tai Chi practitioners (Posner et al., 2007; Xue et al., 2011; Craigmyle, 2013; Guleria et al., 2013; Wei et al., 2014). Our results confirmed our hypothesis and suggest that a potential mechanism through which combined physical and mental exercise may favor cognitive performance is by facilitating early attentional processing in parieto-frontal networks.

## Materials and Methods

### Participants

**FIGURE 1:**
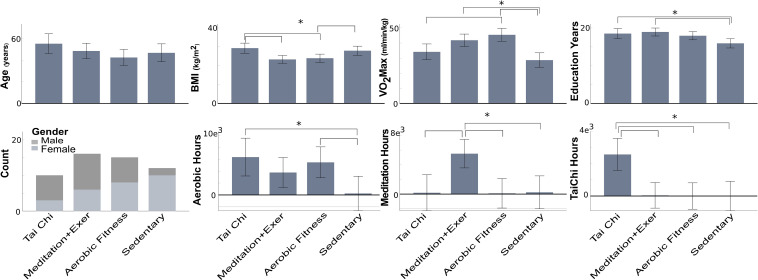
Participant features by group disciplines. Mean and confidence interval at 0.95 by feature. VO_2_ Max, estimated maximal oxygen uptake; BMI, body-mass index; Asterisk with brackets index: significant group differences (*p* < 0.05), the error bars represent the confidence interval.

Fifty-three participants from the primary study (Hawkes et al., 2014a) living independently without neurological or physical disorders were included. The original study recruited participants with a wide age range (22–75) as one of the original aims was to study the effect of aging on cognitive performance. However, most of the participants belonged to the middle age range (44–65 years). The mean of age by groups is displayed in Figure 1, showing no differences between groups. The final sample for each group in this secondary data analysis was, Tai Chi (TC), *n* = 10; meditation + exercise (MEDe), *n* = 16; aerobic exercise (AER), *n* = 15; sedentary (SED), *n* = 12.

Subjects were recruited by flyers posted within Eugene, Oregon, online craigslist ads, and public service lectures on exercise as medicine in Eugene, Oregon area during the 2010–2011 years. All subjects were normally aging volunteers from Lane County, Oregon in which the city of Eugene is located. Health regimen practitioners (TC, MEDe, and AER) were required to have practiced at least 5 years or more, three times/week, 30 min/session. The commitment to the discipline was documented for all participants by means of a self-report questionnaire with number of days per week and minutes per session, as well as years they had practiced Tai Chi, meditation or aerobic activities. Sedentary participants were required to have been sedentary for five or more years, with no prior experience with meditation or Tai Chi. Figure 1 indicates the number of hours practiced by participants, at the moment of the study, to either aerobic exercise, meditation or Tai Chi. The aerobic activities practiced by participants included, jogging, biking, and hiking. The meditation activities included both concentrative (e.g., focus on the breath) and open-awareness (e.g., practicing bringing awareness to the present moment) practices. Tai Chi training included a variety of styles, including Yang and Chen style Tai Chi. Physical + mental groups were considered (1) Tai Chi, because it integrates a variety of movement types, breath, and cognitive skills, including focused attention, imagery and multi-tasking; and (2) meditation + exercise, because meditators performed both the executive attention practice of focusing on the breath or keeping attention on the present moment and one of the aerobic exercises described above.

The University of Oregon Institutional Review Board approved the primary study. Written informed consent was obtained from all subjects. For more details see Hawkes et al. (2014a,b). Data analyzed in this study was de-identified.

### Executive Attentional Task

**FIGURE 2:**
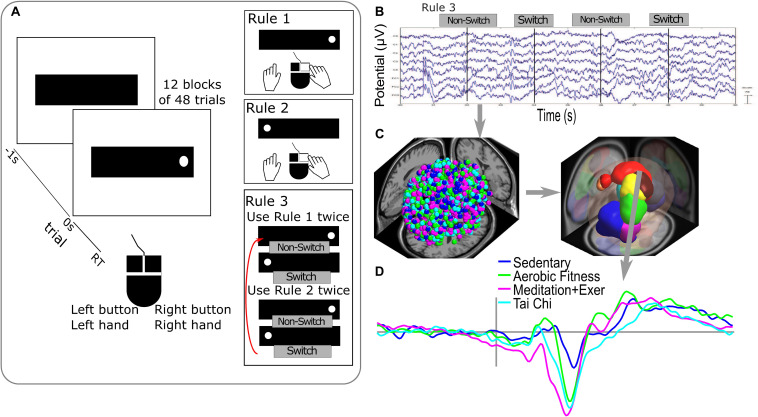
Executive attentional task and EEG analysis. **(A)** Visuospatial switching task paradigm used in the study that consists of different rules during the responses with a two-button mouse. Rule 1 required pressing the mouse button on the same side as the dot location on the screen (time 0 s). Rule 2 required pressing the button opposite to the dot’s screen location. Rule 3 required subjects to switch between rule 1 and 2 on each 2 trials. **(B)** Epochs of raw EEG signals used in the analysis were the correct responses for switch and non-switch trials from Rule 3 blocks. **(C)** Processing of EEG signals in sources dipoles (on the left side, dot colors represent all the dipoles obtained colored by groups) and clustering brain EEG sources dipoles in Brain Domains by similarities in location and ERP activity (measure projection domains are represented by colors blobs detailed in Table 1). **(D)** Example of ERP activity associated with one brain domain (superior frontal) representing the average by group in the color lines.

Subjects performed a randomized alternating runs, non-cued visuospatial task switch test (VSTS) developed at the Mayr Laboratory, University of Oregon (Mayr, 2001). A computer display located 24 inches in front of the participant showed a red dot in a horizontally oriented fixation rectangle. Each subject was trained to respond as quickly and accurately as possible to stimulus appearance using a two-button mouse (Figure 2A). Rules 1 and 2 trained subjects to respond accurately to Rule 3, the task switching condition. Rule 1 required pressing the mouse button on the same side as the dot location on the screen. Rule 2 required pressing the button opposite to the dot’s screen location. Rule 3, the condition evaluated in Hawkes et al. (2014a,b) and this study, required subjects to switch back and forth between rules 1 and 2 on each 2 trials (e.g., 1, 1, 2, 2, 1, 1, etc.). Thus, rule three comprised switch and no-switch trials which were used to calculate a behavioral index of the switching capacity of the participants as described below. Thus, the same trials were used as in the original ERP analysis, providing an index of the performance on the switch and no-switch conditions in Rule 3 and local switch costs, a normalized measure of the switching capacity from a behavioral response perspective. Visual error feedback was given to the participants, who continued with the task after they corrected their error. Twelve blocks of 48 trials each comprised the task switch condition (Rule 3). The test was programmed in E-Prime (Psychology Software Tools).

### Participants’ Physiological and Behavioral Performance Measures

Body mass index (BMI, body mass divided by height squared) and cardiovascular capacity (VO2max, estimated using the Rockport 1 mile walk) (Kline et al., 1987) are reported in Figure 1. Executive performance was evaluated with the VSTS task using percent local switch costs—calculated by subtracting non-switch (rule 3) from switch (rule 3) reaction time (RT), divided by non-switch (rule 3) RT (Figure 2). Local switch cost is a normalized measure that quantifies the difference between switch and non-switch trials for each group, while controlling for the speed/accuracy trade-off. Thus, this measure is a proxy for the ease with which switching between tasks occurs at the neural and behavioral levels. For more details see Hawkes et al. (2014a,b).

### Electroencephalographic Acquisition, Preprocessing of EEG Data and ERPs

In the primary study, continuous electroencephalography (EEG) data were recorded in a Faraday cage protected room with a 256-electrode Electrical Geodesics (EGI) EEG System 300 and digitized with a 24-bit A/D converter, collected at 250 Hz (EGI, Eugene, OR, United States). The impedance of each sensor was <5 KΩ. Channels were referenced to reference signal (VREF vertex) channel. Subjects were provided with a Table Clamp chin rest.

For this secondary analysis, EEG data were processed using the EEGLAB toolbox (Delorme and Makeig, 2004) in Matlab (The MathWorks, Inc.). Continuous data were filtered between 1 to 100 Hz; additionally, a notch filter was applied at 60 Hz to remove line noise. Independent Component Analysis (ICA) was performed on 128 of the 256 original channels to improve the quality of the ICA decomposition (Onton et al., 2006). We used the GSN-HydroCel-128 sensor position to select the 128 electrodes included in the ICA.

Data sets were automatically cleaned using the EEGLAB function clean_rawdata^1^, with the following parameters, arg_flatline = off; arg_highpass = [0.25 0.75]; arg_channel = 0.8; arg_noisy = 4; arg_burst = 5; and arg_window = 0.3. Please see the limitations section of the discussion for possible limitations to the arg_burst preprocessing parameter. These multiple algorithms removed low-frequency-drifts, noisy channels, short-time burts and incompletely repaired segments from the data.

The ICA algorithm used was CUDAICA (Raimondo et al., 2012), applied to continuous data to decompose it into source-resolved activities. Then, epochs were created using a time window of 3, −1.5 to 1.5 s, with respect to the red dot onset of the VSTS task (Figure 2A). Epochs with artifacts in specific channels were removed using an EEGLAB automated method based on extreme values of potential, data improbability and potential kurtosis (Delorme et al., 2007).

The selected epochs for the analysis were 90 epochs for the correct switches on Rule 3 (Rule 3 required subjects to switch back and forth between rules 1 and 2 every two trials, called Switch) and 90 epochs for correct answers on non-switch trials on Rule 3.

**FIGURE 3:**
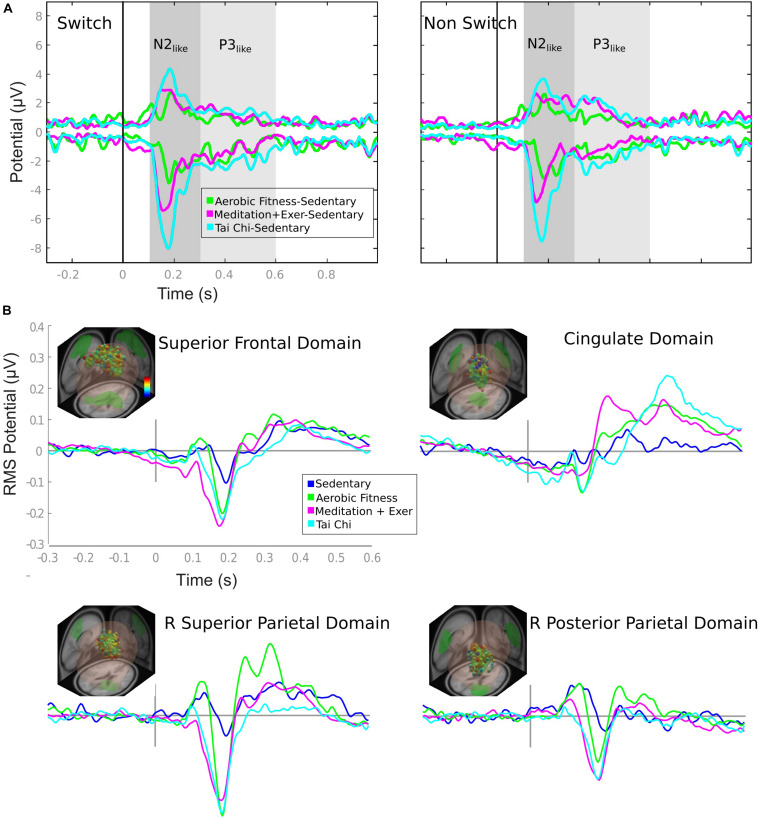
ERP envelopes by group comparison and domain ERPs by group. **(A)** ERP envelopes in 1 s after stimulus presentation, contrasting active groups against sedentary group. The colored lines represent the subtraction showed in the legend. Gray shaded areas represent the time window considered for the ERP comparison of Figure 5. **(B)** Each domain contains the information of: location (e.g., superior frontal domain) (R, right; L, left); representative dipoles with colored dots related to a color bar with the probability of belonging to the domain (from 0 [blue] to 1 [red]); and ERP activity of switch epochs is in colored lines matched to the group legend (Sedentary to Tai Chi).

Envelopes of the ERP differences were visualized in order to identify the time windows containing the greatest ERP differences between groups. ERP envelopes were calculated using a (2 × time points) matrix whose rows represent the most positive and negative value, of all channels, at each time point. The sedentary group was contrasted with each of the active groups (TC, MEDe and AER) in the time window between −0.3 and 1 s. A first time window between 0.1 and 0.3 s showed the more evident differences and a second time window between 0.3 and 0.6 s revealed more subtle differences (Figure 3A). The ERP envelopes *per se* do not indicate the sources or brain regions generating the differences. Thus, further source-resolved ERP analysis was performed. In order to estimate the brain sources contributing to the differences depicted by the envelope of the ERP difference, we performed an estimation of equivalent current dipole locations using a Boundary Element Model of Montreal Neurological Institute (MNI) head model with the EEGLAB dipfit plugin (DIPFIT^2^). Dipoles were then clustered in brain sources with the methods explained in the next section. ERPs were estimated for each brain source identified in the cluster analysis. In order to statistically explore the differences visually detected with envelopes, a time window from −0.3 to 0.6 s was used, corrected with a baseline from −0.3 to 0 s, with the polarity corrected by topoplots, filtered below 30 Hz and then averaged across data trials.

### Clustering of Independent Components Dipoles

The clustering method used in this study determined the similarities between subjects, by conditions and groups. Correct responses for switch trials (transitions from the congruent to incongruent instruction and vice versa) and non-switch trials from Rule 3 blocks (Figure 2B) were analyzed. The Measure Projection Toolbox (MPT)^3^ (Bigdely-Shamlo et al., 2013), was used to cluster brain dipoles from all subjects (Figure 2C). The MPT uses a template brain space grid of voxels of 8 mm (here MNI). Each voxel receives a probability of a representative ERP activity and location from the dipole information. To get the spatial domains - or probable location of the source-resolved activity—MPT clustered the brain subspace based on the correlation between ERPs of dipoles in nearby locations (Figures 2C,D).

The parameters used were a correlation threshold of 0.9 with a *p*-value less than or equal to 0.05. To increase the robustness of the results, measure projection analysis (MPA) was applied to 2,000 surrogate data with a false discovery rate (FDR) correction, resulting in a final *p*-value threshold less than or equal to 0.012. Furthermore, a three-dimensional Gaussian location error was considered in the location estimation. This error was equal to 12 mm with 3 standard deviations (3 6mm).

Finally, we selected the brain domains that had almost all the participants by group (greater than 85% of the participants by group), which resulted in the inclusion of areas related to executive functions previously reported during switching paradigms.

### Participants Features, Behavioral, and ERP Statistics

All the statistics were analyzed in RStudio [RStudio Team (2016). RStudio: Integrated Development for R. RStudio, Inc., Boston, MA, United States]^4^

Participants features (Age, Education years, VO2max, BMI and self-reported training hours) were compared between groups using analysis of variance (ANOVA) with Sidak adjustment as a *post hoc* test (Figure 1).

**FIGURE 4:**
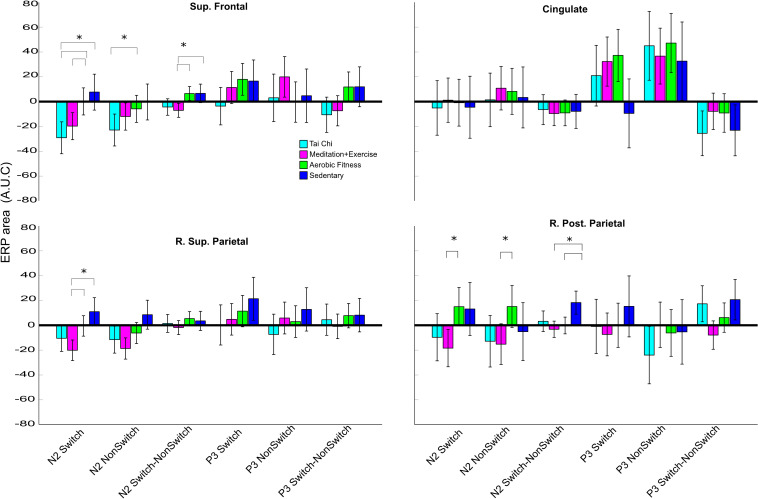
Time-window (N2-like and P3-like area) differences between groups by domains and conditions. Each subfigure represents the average area under curve of N2-like (100–300 ms) or P3-like (300–600 ms) by group per domain and conditions. The conditions are switch trials from rule 3, non-switch from rule 1 and a subtraction of both, shown in the ticks of the X axis. The error bar represents the confidence interval at 0.95. Asterisks show a significant difference (*p* < 0.05) between the group connected by brackets.

Participant behavioral scores and ERP time windows were compared using multivariate analysis of covariance (MANCOVA) with Age, Education years, VO2max and BMI as covariables and group as the predictor and sex as cofactor. For the pairwise comparisons, we used a *post hoc* Sidak adjustment. We analyzed two ERP time windows as the area under the curve (Figures 3A, 4). The first-time window was between 100 and 300 ms after the stimulus presentation (characterizing the N2-like wave in frontal and parietal domains), and the second time window was between 300 and 600 ms (characterizing the P3-like wave in the cingulate domain).

**FIGURE 5:**
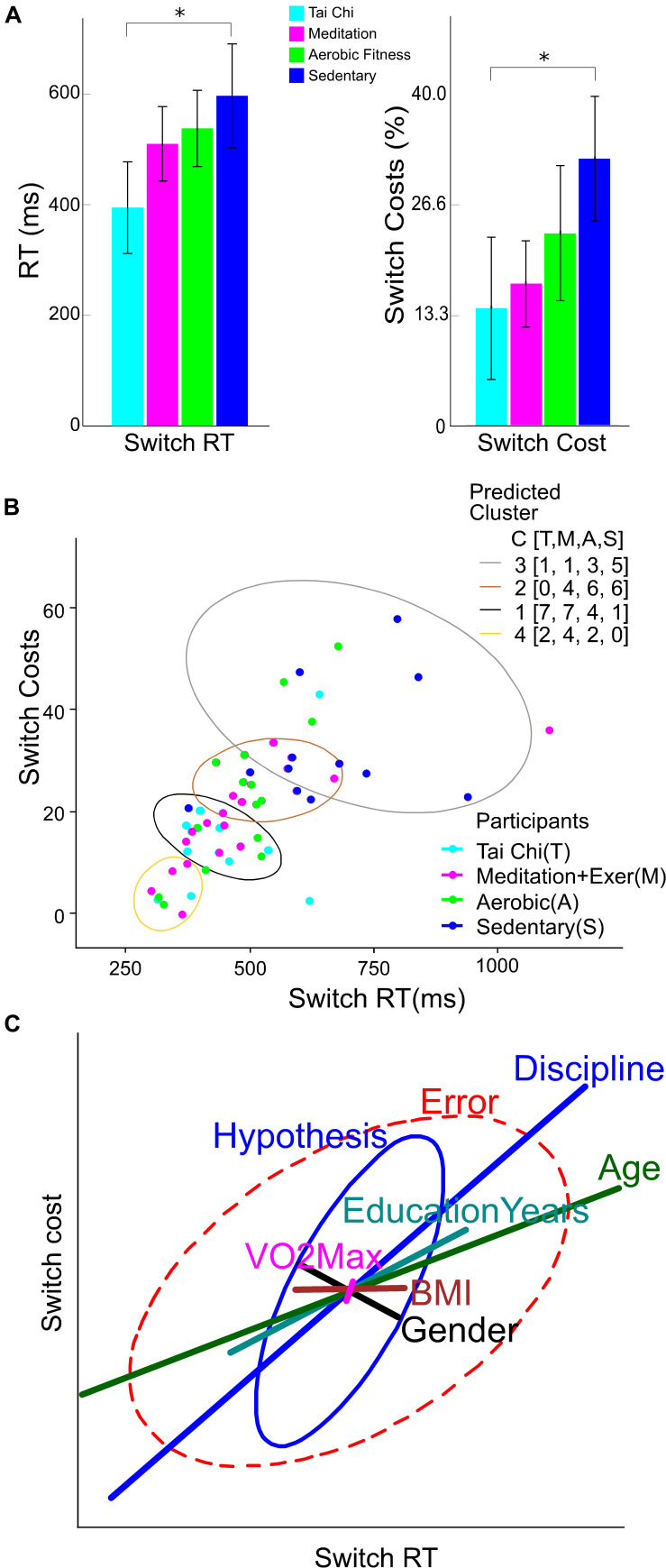
Behavioral performance, clusters and variables weight in switching performance. **(A)** Reaction times (RT) in switch trials (left Y axis), and switch costs (right Y axis) by group discipline. The error bar represents the confidence interval at 0.95. Asterisks show a significant difference (*p* < 0.05) between the group connected by brackets. **(B)** K-Mean clusters of Switch reaction time and Switch cost. The legend labeled as Participants colored by group, light blue for Tai Chi (T), light red for meditation + exercise (M), green for Aerobic fitness (A) and blue for Sedentary participants (S). The legend labeled as Predicted Cluster contain the colors of ellipses that shows the output of participants by cluster number from 1 to 4. Additionally contain a counting of participants by group in C[T,M,A,S]. **(C)** Ellipses represent the hypothesis and error effects of the MANCOVA model and its orientation; the relation between variables showed in the x and y axis. The ellipse length represents the effect size in the corrected model, its width represents the data dispersion. The line length in colors represents the effect of the difference factor and covariables in the corrected model. The line orientation represents the relation between x and y variables.

**FIGURE 6:**
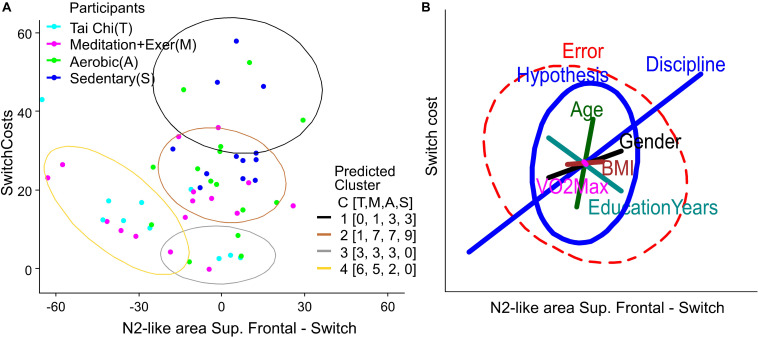
Clusters and variables weight in Behavior-ERP relationship. **(A)** K-Mean clusters of switch cost and N2-like of right superior parietal domain. The legend labeled as participants is colored by group, light blue for Tai Chi (T), light red for meditation + exercise (M), green for Aerobic fitness (A) and blue for Sedentary participants (S). The legend labeled as Predicted Cluster contains the colors of ellipses that show the output of participants by cluster number from 1 to 4. Additionally, it contains a counting of participants by group in C [T,M,A,S]. **(B)** Ellipses represent the hypothesis and error effects of the MANCOVA model and its orientation, with the relation between variables shown in the x and y axis. The ellipse length represents the effect size in the corrected model, its width represents the data dispersion. The line length in colors represents the effect of the difference factor and covariables in the corrected model. The line orientation represents the relation between x and y variables.

To visualize the nature and dimensionality response variation of the predictor (Group), cofactor (sex) and covariables (Age, Education years, VO2max and BMI) in the MANCOVA model we use heplot R function^5^. This function plots ellipses representing the hypothesis and error sums-of-squares and products matrices for terms and linear hypotheses in a multivariate linear model (Figures 5C, 6B).

Finally, to visualize the variables without the influence of the group discipline factor we computed a K-Means clustering (with 4 expected groups) for the behavior using the Switch cost and Switch RT variables, and for a relation Behavior-EEG using Switch cost and the N2-like-area from right superior parietal domain (with the strongest group differences in MANCOVA). Then we contrasted the predicted cluster with the participants by groups, counting them in each predicted cluster (Figures 5B, 6A).

## Results

### Group Variables Comparison

Here we provide group contextual variables that inform our interpretation of the neural results, including age, education years, VO_2_max, BMI, and self-reported hoursc of practice (Figure 1).

There were no significant differences in age between groups. The Tai Chi (TC) group had higher BMI compared to the Meditation + Exercise (MEDe) and Aerobic fittness (AER) groups (*p* = 0.005 and *p* = 0.017, respectively). The Sedentary (SED) group had higher BMI than the MEDe group (*p* = 0.033).

The SED group had lower VO_2_max than the AER and MEDe groups (respectively *p* < 0.001, *p* = 0.001). The TC group had lower VO_2_max than the AER group (*p* = 0.009).

The SED group had fewer education years than the MEDe (*p* = 0.003) and TC groups (*p* = 0.036).

With respect to sex, the MEDe and AER groups were similar in terms of the number of males and females, but the TC group had more males, and the SED group had more females.

Self-reported practice of TC, MEDe, and AER regimens showed TC practitioners reported significantly higher TC practice than the other three groups (MEDe, AER, SED, *p* = 0.002, *p* = 0.003, and *p* = 0.004, respectively), MEDe reported significantly higher meditation practice than the other three groups (TC, AER, SED, *p* = 0.002,*p* = 0.002,*p* = 0.004, respectively), and AER reported a similar amount of aerobic exercise compared to TC practitioners and MEDe, but a significantly greater amount of aerobic practice compared to SED (*p* = 0.021) (Figure 1).

In summary the SED group showed worse physical condition, fewer education years and training hours as expected; however, they were similar to the TC group on BMI and VO_2_max.

### Performance on the Executive Attentional Task

When comparing the performance on the attention switching task for the four groups, corrected only for Age, it was found that the Tai Chi and Meditation plus exercise groups showed significantly faster switch RT than the SED group (TC *p* < 0.001, MEDe *p* = 0.001, AER *p* = 0.026). The percent local switch costs showed with similar results, except for the AER group (TC *p* = 0.001, MEDe *p* = 0.006, AER *p* = 0.446).

However, when the comparison between groups was corrected for Age, Education years, VO_2_max and BMI as covariables and sex as cofactor (Figure 5A), results showed that only the TC group had faster switch RT than the SED group (TC *p* = 0.010, MEDe *p* = 0.714, AER *p* = 0.936). In addition, percent local switch-costs were significantly smaller only for the TC group compared to SED (TC *p* = 0.043, MEDe *p* = 0.464, AER *p* = 0.987). This attention efficiency proxy (switch cost) was not statistically different between the TC and MEDe and AER (*p* = 0.863 and *p* = 0.164, respectively), and the AER was not statistically different from the SED group (*p* = 0.982).

Figure 5B shows that SED participants (blue dots) differed on switch costs and switch reaction times from all the other disciplines, whereas TC participants had better performance with less data dispersion (light blue dots) compared to MEDe and AER participants. The K-means clustering for the four groups showed that most of the people with the poorest performance (higher switch costs and higher switch RT) belong to the SED group (Figure 5B, gray ellipse), the people with the better performance belong to the TC and MEDe groups (yellow and black ellipse), while people from the AER group had an intermediate performance, most of the people in this group are contained in the brown and black clusters (see the legend C [T,M,A,S] of predicted cluster in Figure 5B, indicating number of participants in each cluster by group).

To evaluate the potential influence of Age, Education years, VO_2_max, and BMI on reaction times and switch costs, we performed a heplot to show the MANCOVA results (Figure 5C) contrasting these variables with the predictor (group discipline). Discipline groups and Age had the main and the only significant effect size in the model (Partial Eta Square = 0.293 and 0.301 respectively), where older people tended to perform better, with slower Switch RTs (less effect on switch costs. Years of education also appeared as a positive predictor of performance, but this was not significant (Partial Eta Square = 0.087). The BMI and VO_2_max factor had less influence in the model (Partial Eta Square = 0.029 and 0.002 respectively). Sex as cofactor indicates that gender was not correlated with performance, having a small influence compared to the Discipline factor (Partial Eta Squared = 0.040). Other combined effects in the model such as Discipline-Age or Gender-BMI did not have significant effects. The combined Gender-Age effects showed the smallest non-significant influence (Partial Eta Squared = 0.121).

### Brain Domains

The clustering algorithm showed 11 brain domains involved in the performance of the switch task. The probable location of each domain is shown in Table 1, calculated based on the average location of each dipole within the domain’s MNI brain space. Within the domains revealed by MPA, there were three domains located close to or in the primary visual and motor areas and the visual associative area, and eight domains with probable location in the superior frontal, anterior cingulate, and posterior parietal regions. However, some domains did not show activation in all subjects. Thus, we only analyzed domains that were active in at least 85% of participants per group, resulting in four domains that were subjected to further analysis: domains 1, 3, 4, and 5 (Table 1). Coincidently the brain location probability of these domains are associated with areas of attentional control and executive functions (Petersen and Posner, 2012), indicating that executive attentional network components were activated in all four groups.

**TABLE 1:** Anatomical information by Domains (BA, Brodmann Area; Prob, probability; R, right; L, Left).

	Brodmann Areas			Anatomical Areas	
		
Domains	Area	Prob.	Description	Area	Prob.
**1.- Superior Frontal**	BA 6 BA 31 BA 24 BA 4 BA 3 BA 5	0.42 0.15 0.13 0.09 0.06 0.06	Premotor and Supplementary Motor Primary Motor Primary Somatosensory Somatosensory Association	L Superior Frontal Gyrus R Superior Frontal Gyrus L Precentral Gyrus L Postcentral Gyrus	0.33 0.25 0.14 0.05
2.- Secondary Visual (V2)	BA 18 BA 17 BA 19 BA 30	0.50 0.26 0.14 0.06	Secondary Visual (V2) Primary Visual (V1) Associative Visual (V3)	Cerebellum R Lingual Gyrus L Lingual Gyrus R Inferior Occipital Gyrus R Middle Occipital Gyrus	0.48 0.15 0.11 0.10 0.06
**3.-L Anterior Cingulate**	BA 24 BA 23 BA 32	0.50 0.42 0.07		**L Cingulate Gyrus** L Caudate R Cingulate Gyrus L Superior Frontal Gyrus R Caudate Brainstem	0.33 0.27 0.17 0.08 0.07 0.05
**4.-R Superior Parietal**	BA 7 BA 5 BA 4 BA 31 BA 3 BA 40	0.24 0.19 0.17 0.12 0.12 0.10	Somatosensory Association Somatosensory Association Primary Motor Primary Somatosensory Spatial and Semantic Processing	**R Superior parietal Gyrus** R Postcentral Gyrus R Precentral Gyrus R Supramarginal Gyrus	0.39 0.30 0.15 0.07
**5.-R Posterior Parietal**	BA 39 BA 31 BA 37 BA 22 BA 19 BA 30	0.30 0.16 0.12 0.09 0.07 0.06	Auditory Processing Associative Visual (V3)	**R Angular Gyrus** R Middle Occipital Gyrus R Superior Parietal Gyrus R Middle Temporal Gyrus	0.33 0.25 0.18 0.09
6.-R Anterior Cingulate	BA 24 BA 32 BA 6 BA 33	0.43 0.37 0.08 0.06	Premotor and Supplementary Motor	**R Cingulate Gyrus** R Caudate R Middle Frontal Gyrus L Cingulate Gyrus R Superior Frontal Gyrus R Inferior Frontal Gyrus L Superior Frontal Gyrus L Caudate	0.20 0.17 0.16 0.13 0.10 0.08 0.05 0.05
7.-R Precentral	BA 4 BA 3 BA 6 BA 40 BA 2 BA 5	0.29 0.26 0.21 0.10 0.06 0.06	Primary Motor Primary Somatosensory Premotor and Supplementary Motor Spatial and Semantic Processing Primary Somatosensory Somatosensory Association	R Precentral Gyrus R Postcentral Gyrus R Superior Frontal Gyrus R Supramarginal Gyrus	0.52 0.27 0.09 0.05
8.- Associative Visual (V3) R	BA 19 BA 37 BA 18	0.42 0.31 0.18	Associative Visual (V3) Secondary Visual (V2)	Cerebellum R Inferior Occipital Gyrus R Lingual Gyrus R Inferior Temporal Gyrus R Fusiform Gyrus R Middle Occipital Gyrus	0.39 0.32 0.09 0.08 0.06 0.06
9.-R Anterior Cingulate	BA 24 BA 32 BA 33 BA 13 BA 47 BA 45	0.34 0.15 0.15 0.13 0.13 0.08	Inferior Insula Pars Triangularis Broca’s Area	R Caudate R Cingulate Gyrus R Middle Frontal Gyrus R Putamen R Middle Orbitofrontal Gyrus R Inferior Frontal Gyrus R Insular Cortex	0.30 0.17 0.15 0.08 0.07 0.06 0.06
10.-L Posterior Parietal	BA 40 BA 39 BA 19	0.49 0.45 0.06	Spatial and Semantic Processing Associative Visual (V3)	L Angular Gyrus L Supramarginal Gyrus	0.70 0.26
11.-L Superior Frontal	BA 10 BA 11 BA 32	0.42 0.30 0.28		L Superior Frontal Gyrus R Superior Frontal Gyrus L Middle Orbitofrontal Gyrus L Gyrus Rectus L Middle Frontal Gyrus R Gyrus Rectus	0.41 0.15 0.13 0.13 0.11 0.07

*Bold font indicates brain domains and their most probable location associated with attentional control and executive functions that are further analyzed in the result section.*

Furthermore, we explored how these domains were involved in the processing of switch and non-switch events for each group.

### Executive Attention Brain Domains Contributions to Channels ERP

The ERP envelopes of the SED group (maximum and minimum voltage from all channels in time) were contrasted against each of the exercise groups for switch and non-switch conditions. All comparisons – TC minus SED, MEDe minus SED, and AER minus SED – revealed that the greatest differences happened between 0.1 and 0.3 s and between 0.3 and 0.6 s (shaded areas in Figure 3A). The ERP waveform for these domains resembled the N2 component (Moore et al., 2012); therefore, we will refer to this waveform as the N2-like ERP. All three aerobic and combined regime training groups showed a positive modulation that resembled a P300. Traditionally the P3 is reported to have fronto parietal sources (Bledowski et al., 2004; Volpe et al., 2007). Our results showed the Anterior Cingulate Cortex as a possible source for this waveform, which has also been shown in other studies (Bledowski et al., 2004; Volpe et al., 2007). Thus, we will refer to this waveform as the P3-like ERP. Statistical differences within these time windows are reported in Figure 4.

The source ERP activity seen in the four brain domains included in the analysis and shown in Figure 3B (only switch trials are shown) explain around 85% (84.1–89.3%) of the variability observed in the channels, with the superior frontal and parietal domains accounting for most of the variability in all groups.

### N2-Like and P3-Like ERP Differences: Controlling for Covariates

In the behavioral results section, we described how age and years of education contributed to the behavioral differences. Thus, it is necessary to investigate how much of the source ERP differences are explained by our main predictor Group Discipline, with Age, Educations years, VO_2_max and BMI as covariables, and sex as cofactor. Thus, we used a multivariate analysis of covariance (MANCOVA), comparing N2-like and P3-like ERP modulations, and we used the area under the curve (AUC) as an index of the ERP amplitude. We included three conditions for the ERP comparisons: (1) Switch, (2) non-Switch and (3) its difference (Switch-Non-Switch). The difference between switch and non-switch was calculated to represent a neural estimation of switch costs or a normalized representation of switch trials. The analysis was performed separately for the main ERP domains (superior and posterior parietal, frontal, and cingulate domains shown in Figure 3B).

We will describe the results in three subsections, to clearly report how aerobic only or combined aerobic plus mental regimes affect neural processing during an attention switch task: (1) First, we describe whether a combined mental-physical training program or an aerobic regime showed any differences over a SED regime alone. (2) Then we describe the difference in combined mental-physical training over physical training alone. (3) Finally, we report ERP differences between the TC and MEDe groups. The ERPs by group are shown in Figure 3B and the statistical results are shown in Figure 4.

(1) Physical/mental activity vs sedentarism. In general, the SED group showed the smallest ERP amplitudes across brain domains. The superior frontal, right superior parietal, and right posterior parietal domains showed greater amplitudes within the N2-like time window, 0.1–0.3s, for the three training groups compared to the SED group (Figure 3). Most of the differences seen in the N2-like ERP remained significant after correction for multiple comparisons. Figure 4 shows that the TC group had a greater N2-like ERP negativity for the Switch trials relative to the SED group (*p* = 0.002) in the superior frontal domain. Additionally, in the right superior parietal domain, the MEDe group showed a greater N2-like ERP negativity for the Switch trials compared with the SED group (*p* = 0.003). The MANCOVA also revealed that in the posterior parietal domain, the MEDe and AER groups differed from the SED group (respectively, *p* = 0.004 and *p* = 0.002) in terms of the Switch minus non-Switch difference, whereas in the superior frontal domain only the MEDe group differed from the SED group (*p* = 0.009) for the Switch minus non-Switch difference. Therefore, all discipline groups (TC, MEDe and AER) differed from the SED group in the N2-like ERP and domain. For the P3-like time window, between 0.3 and 0.6s, the most noteworthy difference was observed in the cingulate domain, where the aerobic and combined regime training groups showed a positive modulation that resembled a P3, which has been associated with greater top-down control during an attentional task (Pontifex et al., 2009), whereas this modulation was absent from the SED group (Figure 3B). However, differences in the P3-like ERP did not survive multiple corrections.

(2) Combined Physical/mental activity vs Aerobic exercise. The N2-like ERP began earlier and was more negative for the MEDe and TC groups (Figure 3B) compared to the AER group, across the superior frontal gyrus, right superior, and right posterior parietal domains. The MEDe group had significatively larger N2-like ERP negativity in the Switch condition compared to the AER group in the superior frontal gyrus (*p* = 0.040), right superior parietal (*p* = 0.007), and right posterior parietal (*p* = 0.007) domains. The MEDs group also showed a greater N2-like ERP negativity for the Non-Switch trials relative to the AER group, but only in the right posterior parietal domain (*p* = 0.049). In contrast, the differences between the TC and the AER groups were significant only for the superior frontal domain, for both the switch (*p* = 0.002) and non-switch (*p* = 0.023) N2-like ERPs, with the TC group showing greater negative modulations (Figure 4). The Switch minus non-Switch difference between combined regimes and the AER group was only significant in the superior frontal domain for the MEDe group (*p* = 0.006), which showed greater negativity relative to the AER group. Therefore, even when both combined regimes, TC and MEDe, showed significatively greater N2-like negativities over the AER group, it was the MEDe group that most consistently differed from the AER group across domains and conditions.

(3) Differences between combined regimens (MEDe vs TC). In general, these groups showed similar activations in all executive domains. The parietal domains did not show any difference between these two groups, but some differences were observed in the frontal and cingulate domains. However, none of these differences resulted significant after correction for multiple comparisons (Figure 4).

In summary, (1) the differences between a combined mental-physical training program or an aerobic regime compared to the SED group were reflected in a larger negative amplitude of the N2-like component in the superior frontal and posterior parietal domains (2) The differences between following a combined mental physical regime in relation to a pure aerobic regime were mainly seen in the superior frontal domain, with some significant differences between the TC and AER groups in the parietal domains, with a more negative N2-like waveform for the combined regime groups. Moreover, (3) the differences between both combined regimes were not significant. Finally, it was the Switch condition in the superior frontal domain that showed most the group differences.

### N2-Like ERP Differences: Factoring Out Age, Education Years, VO_2_max, and BMI

In order to clearly report how much of the significant results reported above can be explained by Group Discipline, we are here indicating the effect size of each of the predictors included in the MANCOVA model. The results confirmed that the significant effects we found were mainly explained by the Discipline (Partial Eta Squared < 0.538) which people practiced: SED, AER, MEDe, or TC. Also, BMI had a significant influence (Partial Eta Squared = 0.264). Another important influence but with lower effect size was the sex of the participants (Partial Eta Squared = 0.196), Education Years (Partial Eta Squared = 0.092), Age (Partial Eta Squared = 0.228 and VO_2_max (Partial Eta Squared = 0.144) had small influences on the results reported above. The factor Discipline also was a significant predictor of the model when combined with age and VO_2_max: Discipline-Age (Partial Eta Squared = 0.524), Discipline-VO_2_max (Partial Eta Squared = 0.441), as well as the factor Gender combined with BMI (Partial Eta Squared = 0.305). Thus, even though Disciple seems to be the main predictor it is important to take into account the multifactorial variables that can influence cognitive performance when interpreting the current results and how they combine to explain executive attention behavioral and neural outcomes.

### Relationship Between N2-Like ERP and Behavior

Finally, to generate a combined model from Behavior and EEG we performed a K-means cluster analysis (Figure 6A) and produced a MANCOVA model (Figure 6B) based on Switch Costs, as the behavioral variable, and the N2-like ERP area under the curve (AUC) for the Switch trials, as the neural variable. We used the Switch ERPs from the superior frontal domain, because it was there that all the groups showed significative differences (Figure 4, top-left panel).

Figure 6A shows that most of the TC and MEDe participants had lower switch costs with a larger negative N2-like AUC (light blue and purple dots are mostly located within the brown and yellow clusters, respectively). Note that the larger a negative number is on the x-axis the larger is the ERP negativity (see Figure 4 top-left for AUC and Figure 3B for ERP waveforms). In contrast, SED participants showed higher switch costs associated with smaller and more positive N2-like AUC values (blue dots are mostly located around zero or slightly toward positive values). All SED participants were contained within the higher Switch costs clusters (Figure 6A, black and brown ellipses). Also, the cluster containing participants with lower performance (black ellipse), have mainly SED and AER participants and only one participant from the MEDe group. Note that N2-like AUC values close to zero mean that the negative deflection of the N2-like ERP was very small (as shown in Figure 4 top-left and Figure 3B). Similar to the behavioral results, people belonging to the AER group (green dots) showed intermediate values (Figure 6A, brown and gray ellipses). See the legend C [T,M,A,S] of the predicted cluster in Figure 6A, indicating the number of participants in each cluster by group. These results suggest a better switching performance was associated with greater negative N2-like ERPs for most of the participants.

To evaluate the potential influence of Age, Education years, VO_2_max, and BMI on the relationship between switch costs and the N2-like AUC, we show the MANCOVA results in a heplot. Similar to the behavioral results, the model shows that Discipline had the greatest significant effect (Partial Eta Squared = 0.675), followed by Age (Partial Eta Squared = 0.383) (Figure 6B). As previously reported, there was no significant difference in age across groups (Figure 1). Thus, it is unlikely that age alone explains the relationship between Switch Costs and N2-like ERPs. The other variables only had a moderate influence and were non-significant in the model; BMI (Partial Eta Squared = 0.260), sex (Partial Eta Square = 0.209) and Education years (Partial Eta Squared = 0.136). VO_2_max had a smaller influence (Partial Eta Squared = 0.083). Therefore, our analysis indicates that Discipline had the greatest effect on the relationship between attention behavior and N2-like ERPs. Nevertheless, a broader interpretation of these results is required, as the combination of factors also appeared as significant predictors in the model: Discipline-Age (Partial Eta Squared = 0.532), Discipline-VO_2_max (Partial Eta Squared = 0.422) and Gender-BMI (Partial Eta Squared = 0.283), indicating that the combined effects of these factors is important.

## Discussion

The present study investigated behavioral and neural correlates underlying a switch-task paradigm across people who chronically adhered to different exercise disciplines, varying in the degree of physical and mental activity required to execute each discipline. We reanalyzed the results obtained by Hawkes et al. (2014a,b) including a different statistical approach to known confounders and using a source-resolved approach (ICA) that allowed us to describe brain sources for ERPs. In addition, we used a cutting-edge method to perform group analysis (Bigdely-Shamlo et al., 2013), to identify common brain source activations between groups. This approach reduces the risk of equating brain activity produced at different electrode locations. Furthermore, we explored source-resolved ERP activity, instead of the more conventional channel ERP analysis, and applied a time-window statistical analysis. We used a MANCOVA to probe group differences and how much of these differences could be attributed to our main predictor factor: Discipline. As far as we know, there are no previous studies that contrast differences between combined mental and physical training, as Tai-Chi (TC) or Meditation + Exercise (MEDe), compared to aerobic exercise only (AER) or sedentarism (SED) in healthy participants using these methods. Thus, our results indicate there is an additive effect of the combined practice of physical and mental exercise in terms of behavior and its neural correlates based on N2 area under the curve analysis. In addition, we confirmed the participation of brain areas associated with executive functions (frontal, parietal, and cingulate areas) as generators of ERP components previously associated with switching tasks. Particularly, the N2-like component of the Superior Frontal domain appears as the area that better differentiates the different groups. Interestingly, the ERP amplitude in this area (measured as area under the curve) correlated with performance, such that a greater N2-like area under the curve was associated with better performance. The MANCOVA test on switching performance and ERP waveforms revealed that activity type was the most relevant factor, with an important influence of age, educational years, and BMI with small effects of VO_2_max and sex.

### Effects of Exercise Discipline and Confounding Factors

Our results differentiated the benefits of physical and mental regimes compared to a sedentary regime on a demanding non-cued switch task and suggest that the practice of combined physical and mental exercise may confer additive benefits in terms of behavior and its neural correlates. Our initial behavioral analysis comparing the switch trial reaction times and switch costs for the four discipline groups, using only age as a covariate, indicated that TC and MEDe practitioners showed significantly lower switch costs and switch reaction times compared to SED individuals, while those who practiced aerobic activity alone were not significantly different from sedentary adults, similar to previous articles by Hawkes et al. (2014a,b). However, when the analysis was made more stringent by including educational years, VO_2_max, body mass index (BMI) and sex, these differences remained significant only for the TC practitioners. These results indicate that for the MEDe group, other factors, such as educational years and reduced BMI, contributed to their smaller Switch Costs and RT, relative to the SED group. The MANCOVA results indicated that Discipline indeed was the factor that explained most of the behavioral results, however, age also appeared as a significant predictor. As reported earlier, there was no significant difference in age across groups (Figure 1). Thus, it is unlikely that age difference explains the group results. In addition, one could expect older participants to show decreased performance compared to younger ones, however, our results report the opposite, the group with better performance was the one that tended to be older (TC). Years of education also appeared as a positive predictor of performance, but this was not significant. The TC and MEDe groups had significantly more years of education relative to SED controls. However, they did not differ in years of education compared to the AER and they outperformed them. This indicates that years of education alone did not explain the difference between groups. In terms of sex, despite having a different ratio of females to males in each group (TC contained mostly males while SEDs were mostly females), sex as cofactor only had a small influence in relation to group differences, as revealed by the MANCOVA. In addition, there are no reports in the literature indicating differences by sex with respect to local switch costs (Grissom and Reyes, 2019). Therefore, it is very unlikely that sex could explain the group differences reported here. In conclusion, the factor most likely explaining the differences in behavior and neural activation during the task switch test is Discipline. Here, we would like to point out that our participants had a middle age range (44–65 years) that from the point of view of the hypothesis of adaptive brain capacity (Raichlen and Alexander, 2017), suggests that early lifestyle changes that include combined physical and cognitive activities can protect the brain structure and function during normal aging, thus can allow participants to mitigate symptomatic neurodegenerative processes. We agree with the mechanism underlying this hypothesis, that a combination of simultaneous exercise and cognitive engagement acts as a stimulus for maintaining brain capacity across the lifespan (Raichlen and Alexander, 2017), and it is likely that combined physical and mental disciplines (TC and MEDe) confer benefits to cognitive performance (more neural specific mechanisms for the difference between groups are discussed below). One might ask if a minimum of a 5-year training time period is reasonable to use in this research study based on the time the brain needs to reorganize attentional activation patterns in response to mental and physical activity programs. In fact, a number of studies have shown significant brain reorganization and structural changes in response to as little as 8 weeks of mental (see Gotink et al., 2016 for a review) or physical training (see Smith et al., 2010 for a review). Thus, 5-years would be enough to produce a behavioral and neural effect.

### Active (Physical or Mental) Regime Over a Sedentary Regime: N2-Like and P3-Like

The SED group had the slowest switch RT and the highest switch costs compared to the three exercise groups, and mainly differed from the TC group as seen in the high dispersion of behavioral data in the MEDe and AER groups. This suggests there are benefits to cognitive performance if normally aging adults engage in any active exercise regimen or combined regimes compared to a generally sedentary lifestyle. But, what about the neural correlates associated with these behavioral results?

Greater N2 and P3 amplitudes have been reported in studies investigating exercise regimes, with some exceptions (Gajewski and Falkenstein, 2015). Hillman and colleagues compared young and older, active and sedentary adults and found larger midline P3 amplitudes in active subjects (Hillman et al., 2006). Tsai and colleagues found greater P3 amplitudes in older adults after aerobic and strength training acute exercise bouts as well as longer term interventions (Tsai et al., 2018, 2019). Similar results were also observed for longer term interventions comparing training in open skills which require more cognitive processing, like table tennis, to closed skills, like aerobic walking or bicycle riding (Tsai et al., 2017). Hawkes et al., 2014b reporting from the same data set observed reduced P3 amplitudes for the SED group. Our source analysis suggested that the most likely candidate to produce this difference was the anterior cingulate domain, however, without significant group differences. Usually, P3s are reported in parietal areas (Bledowski et al., 2004; Volpe et al., 2007), but some studies have also found this type of activity in the cingulate (Volpe et al., 2007; Malinowski et al., 2017). That is why we refer to this modulation as P3-like—however, in this more stringent analysis that included more covariables we did not find significant differences in the cingulate area nor in the P3-like amplitudes. Thus, with our analysis we could not replicate previous studies reporting P3 differences between groups.

In contrast, The N2-like ERP modulation was the one that more consistently showed differences across groups. In terms of the likely brain generator for the N2-like ERP modulation reported here, we found that the superior frontal and parietal cortices contributed to most of the variation in the time window of the N2-like ERP. Accordingly, the source-ERP associated with these brain domains showed clear negative modulations around 200 ms. This is in line with previous literature showing N2 activity in frontoparietal electrodes (Hsieh et al., 2014; Tarantino et al., 2016).

We observed that the SED group showed the smallest and most positive ERP amplitude around 200 ms compared to the exercise groups. This difference occurred in the superior frontal superior parietal and posterior parietal domains. The ERP waveform of these brain domains resembled the N2 component (Moore et al., 2012), which is associated with greater task-related attention or novelty (Luck, 2005). The N2 have been reported in other studies using task switch paradigms (Wylie et al., 2003; Hsieh and Liu, 2008; Gajewski and Falkenstein, 2015; Tarantino et al., 2016); however, the N2 reported in the current study was larger than that seen in previous studies. Accordingly, the greater N2 negativity we found for exercise disciplines is similar to those reported in studies comparing active with inactive individuals (Polich and Lardon, 1997; Pontifex et al., 2009; Taddei et al., 2012): one previous study of combined mental and exercise practice (Mindfulness Training Based Stress Reduction TM [MBSR]) compared to untrained controls using an emotional Stroop task reported differences in N2 amplitude between groups (Malinowski et al., 2017). Taddei et al. (2012) showed that N2 and P3 amplitudes in a Go-no Go task were larger in fencers (which is a physical activity with a cognitive component) vs. inactive controls and this effect was independent of age. Greater ERP amplitudes may be indicative of increased local synchronization of neuron fields in sources which are modulated by attentional processing. This suggests that maintaining an active physical or mental training regime compared to a sedentary lifestyle may provide benefits to early attentional processing of task stimuli and in the decision-making process. Overall, our results suggest decreased top-down activation in the parietal and frontal domains for the SED group during this visuospatial attentional task.

### Combined Physical and Mental Activity Over a Pure Aerobic Regime: N2

In terms of the behavioral results, percent local switch costs and switch RT were not different for MEDe and TC practitioners compared to the aerobic group. However, our K-Means clustering analysis clearly showed that AER participants had an intermediate performance relative to combined regimes and SED people (Figure 5B, black and brown ellipses).

In terms of the neural correlates, we showed that both combined regimes, TC and MEDe, showed significantly greater N2-like negativities compared to the AER group. However, it was the MEDe group that most consistently differed from the AER group across domains and conditions. Also, ERPs for the TC group in the superior frontal domain differed from the AER group but not from the MEDe group. This suggests complex executive function was more efficient in combined regimen practitioners, but depending on the specific exercise regime, different brain areas may account for the differences reported. Thus, we provide the evidence suggesting that combined practice provides an additive effect. What kind of mechanisms are considered to be trained in the combined programs that are needed for switching tasks? Research suggests that the executive attention system is important for task-switch paradigms (Watanabe et al., 2013; Schneider, 2015). This is because the executive system needs to keep track of both the trial sequence, and when in the sequence one needs to switch task rules. The combined program gives not only physical training, which brings more blood to the brain, which has been shown to improve executive function (Guiney and Machado, 2013), but it also trains executive attention circuits through tasks requiring high degrees of focused concentration (Taren et al., 2017). Thus, the combined program improves executive attention in a two-pronged way, through both physical and mental training. A likely neural correlate of the improved performance is the greater N2-like ERP shown by TC and MEDe. Similar to what we discussed in the previous section, the increased N2 amplitudes in the right posterior parietal area may reflect greater top-down control during an attentional task, which allows better executive control and faster responses. Further, Switch costs and N2-like amplitudes showed a positive correlation as shown in Figure 6B.

The MANCOVA model based on switch Costs and the amplitude of the N2-like ERPs during the Switch condition for the superior frontal domain showed that a better switching performance was associated with greater negative N2-like ERPs for most of the participants. The most likely predictor of this correlation is Discipline; however, the combination of Discipline with Age and Physical condition (VO_2_max) also accounted for part of the effect described above. Thus, it is important to interpret these results in the context of multiple factors that affect health, cognitive performance, and general wellbeing in people. Our results are in line with previous studies that suggest the advantage of practicing combined physical and cognitive training compared to physical activity alone (Anderson-Hanley et al., 2012; Barcelos et al., 2015; Eggenberger et al., 2015; Tsai et al., 2017, 2018). For example, Tsai et al. (2017) found that when comparing training in open skills which require more cognitive processing, than closed skills, the open skill training produced reaction time facilitation in switch and non-switch trials, while the closed skill training did not (Tsai et al., 2017).

### Differences Between Combined Regimes

The combined regimens showed small timewise differences in the early stimulus processing time window in frontal and cingulate domains. However, no significant differences were found. These data suggest similarities in the neural processing of attentional information between the two combined training groups.

### Brain Source Analysis and Selection of Brain Domains

The use of a brain source analysis increases the spatial resolution of the EEG analysis (Akalin Acar et al., 2013). However, it is still far from the resolution of spatial information revealed by imaging studies. In the following paragraphs, we will discuss our results in light of evidence from previous imaging studies.

The group analysis method used in this study allowed us to confidently compare the electrical activity produced by the same brain regions by group. Main areas for the switch ERPs were localized to the bilateral superior-frontal, bilateral anterior cingulate (ACC), and right superior, and right posterior parietal cortices. All these brain regions have been described as relevant for the performance of switch paradigms by previous neuroimaging research (Rushworth et al., 2002; Swainson et al., 2003; Brass and von Cramon, 2004; Brass et al., 2005). However, we did not find group differences in the ERP analysis for the cingulate domain and the area that most consistently showed differences between groups was the superior frontal domain. This is relevant because the prefrontal activity in a switching task probably reflects the ability to deal with multiple cognitive rules and to coordinate behavior according to these rules (Nyhus and Barceló, 2009).

The parietal domains were mainly responsible for differences between the MEDe and AER groups (effect of combined regime over a pure aerobic one). The Superior Parietal Domain (SPC) has been recently associated with visuospatial working memory tasks and is especially involved in the manipulation and rearrangement of information that it is necessary during switching trials (Koenigs et al., 2009). Also, the SPC has been associated with visual perception changes during bi-stable paradigms (Kanai et al., 2011) which is similar to the visuospatial processing required to perform the task switch paradigm used in the original study. These results suggest a superior parietal contribution to the speed of processing associated with the rule updating required to perform switch trials and this may be the particular top-down mechanisms by which MEDe performed better than AER. In addition, the Posterior Parietal domain (PPC) has long been studied in relation to attention, and previous studies have shown it contributes to switch task performance and the ability to shift attentional sets (Liston et al., 2006). The right PPC has been associated with the orientation of visuospatial attention toward salient features and with a bottom-up strategy to attend to retrieved memories (Seghier, 2012). In our task, the PPC area could have contributed to differentiating between rules during the switching (N2 area results) and between left and right stimuli during the trials (Hirnstein et al., 2011; Chen et al., 2012). Differences between MEDe and AER in this area suggest that the PPC mediates the additive effect of combined physical and mental disciplines.

### Limitations

The original study recruited subjects from one county in Oregon, United States who fulfilled the requirement of practicing the different regimes (or being sedentary) for more than 5 years. This means causation cannot be established based on exercise practice by exercise type. Further, only the key variables known to explain exercise variance were included in the original study as dependent variables in order to document their association with executive function. Further, training quality, which could partially explain the differences seen between our groups was only evaluated through long-term training effects on VO_2_max and BMI. We used these factors as cofactors in our MANCOVA analysis and showed that the main factor that explained ERP differences was group, over age, VO_2_max, and sex ratio, which is what was seen in the original analysis. Further studies should include measures of depression and mental status, a balanced sex ratio for each group, blood pressure, smoking and drinking status, and nutrition to further document the effects of diverse variables known to affect human cognition, as well as long-term exercise training, on normally aging adult executive function. Additionally, longitudinal training studies comprising the variables above will provide clarity on which variables or combination of variables predict better executive function in normally aging adults.

Also, we tried to include the self-reported hours of meditation, TC, and aerobic activities in the MANCOVA analysis, but the effect size of these variables was close to 0 and also the correlation values (Pearson) with switch cost performance were below 0.2. This could be due to the retrospective report of practice hours not well reflecting the intensity and quality of the commitment or an over or under estimation of the real amount of practice.

The value used in the arg_burst parameter of clean raw_data function (5 standard deviations) during the EEG preprocessing was based on the parameters suggested in Mullen et al., 2015. However, new empirical results (Chang et al., 2020) suggest that a number in the range of 20–30 standard deviations should be used. 5 SD can be aggressive and lead to loss of brain information. Future research may use Chang et al. article as a guideline.

The Measure Projection Analysis used to cluster brain dipoles is a new tool that requires further theoretical and empirical development to provide a more insightful and quantitative interpretation of results. One possible limitation in using this method is known as “double dipping”: MPA first uses ERP measures (in the case of the current study) to create consistent domains and then tests their differences across conditions, using the data twice. Circular analyses have been flagged in fMRI studies (Kriegeskorte et al., 2009; Vul et al., 2009). Unfortunately, to the date of this publication there are neither analytical nor empirical analyses that quantify the impact of this issue in MPA. We strongly believe that MPA is still a valid approach, first, because it is expected that ICs localized within a certain region naturally show correlated activation patterns, even without using the constraint of similarity of measures. Secondly, the number of ICs is orders of magnitude smaller than the number of voxels in fMRI studies, where circular analysis has been described. To increase robustness of our results we have applied MPA to 2,000 surrogate values at each voxel, with a resulting final *p*-value threshold less than or equal to 0.01. Nevertheless, future methodological studies should clarify the real impact of circularity using MPA. Furthermore, even though MPA is more data-driven than other clustering methods, it still requires parameters that are user-specified: local convergence value and maximum correlation value. There are no empirical studies that provide practical guidelines for choosing these parameters. It is a probabilistic approach; thus, neighboring domains can overlap, making interpretation challenging. Thus, there is no absolute certainty for source localizations. In addition, as far as we know, whether the assignment of brain activity to subcortical structures reveals actual deep-source or broad-surface-source activity has not been addressed in methodological studies. It is possible that the use of single subject models to represent brain sources can reduce the accuracy of deep source localization (Nunez et al., 2006). The source localization problem is, however, inherent to any source modeling and clustering method developed so far. Thus, caution is required when attributing brain activity to brain regions and, in particular to deep structures (as is the case of the ACC). For this reason, in this paper, we talk about the probable location of brain domains, rather than brain cortex, regions, or areas. Finally, longitudinal, carefully controlled training studies are needed to further identify brain regions active during executive function tasks in normal adult humans.

### Conclusion

This study is one of a growing number of studies investigating combined physical and cognitive training benefits, including the development of new training-based interventions to delay, mitigate, or eliminate dementia and cognitive impairment. Based on a robust methodology for analysis of brain clustering and multiple comparison between groups with covariables and cofactors, we showed that there is a linear correlation mainly explained by training Discipline, where sedentary people showed the lowest performance, Tai-Chi and Meditation + exercise practitioners showed the better performance and most aerobic-only practitioners showed intermediate performance. Furthermore, performance was correlated with the amplitude of the N2-like ERP negativity, particularly in the Superior Frontal brain domain. These results suggest that combine physical and cognitive activity (such as TC and MEDe) may offer benefits over performing aerobic exercise alone, to improve the executive attention network in healthy aging adults. We hypothesize that at a neural level this benefit is mediated by better early attentional processing of task stimuli. In addition, the importance of other cofactors (such as age and years of education) cannot be ruled out, due to the cross-sectional nature of our study. Thus, future experimental studies and longitudinal designs are needed to confirm the advantage of combined programs and understand the mechanisms that contribute to the cognitive improvements associated with these programs.

## Data Availability Statement

The raw data supporting the conclusions of this article will be made available by the authors, without undue reservation.

## Ethics Statement

The studies involving human participants were reviewed and approved by The University of Oregon Institutional Review Board. The patients/participants provided their written informed consent to participate in this study.

## Author Contributions

PB contributed to the conception, design, and interpretation of the data, drafting, revising, and approval of the work and is accountable for all aspects of the work. GC contributed to the analysis, interpretation of data, critical revision and approval of the work and is accountable for all aspects of the work. TH contributed the original data set and critically revised the manuscript and is accountable for all aspects of the work. IR-S contributed to critical revision and approval of the work and is accountable for all aspects of the work. MW contributed to the conception, design, and interpretation of the data of the work, and revised, approved and is accountable for all aspects of the work. All authors contributed to the article and approved the submitted version.

## Conflict of Interest

The authors declare that the research was conducted in the absence of any commercial or financial relationships that could be construed as a potential conflict of interest.

## Footnotes

1 https://sccn.ucsd.edu/wiki/Artifact_Subspace_Reconstruction_(ASR)

2 http://sccn.ucsd.edu/eeglab/dipfittut/dipfit.htmlold

3 http://sccn.ucsd.edu/wiki/MPT

4 http://www.rstudio.com/

5 https://rdrr.io/cran/heplots/man/heplot.html
